# Improved chondrogenic performance with protective tracheal design of Chitosan membrane surrounding 3D-printed trachea

**DOI:** 10.1038/s41598-021-88830-3

**Published:** 2021-04-29

**Authors:** Hyeonji Kim, Jae Yeon Lee, Hyeonseok Han, Won-Woo Cho, Hohyeon Han, Andrew Choi, Hyeonjun Hong, Jae Yun Kim, Jeong Hun Park, Sun Hwa Park, Sung Won Kim, Dong Sung Kim, Dong-Woo Cho

**Affiliations:** 1 Department of Mechanical Engineering, Pohang University of Science and Technology (POSTECH), 37673 Pohang, Gyeongbuk South Korea; 2 Department of Companion Animal Health, Daegu Haany University, 38610 Gyeongsan, Gyeongbuk South Korea; 3 School of Interdisciplinary Bioscience and Bioengineering (POSTECH), 37673 Pohang, Gyeongbuk South Korea; 4 Institute of Convergence Science, Yonsei University, 03722 Seoul, South Korea; 5 Department of Biomedical Engineering, The Wallace H. Coulter, Georgia Institute of Technology and Emory University, Atlanta, GA USA; 6 Department of Otolaryngology and HNS, College of Medicine, The Catholic University of Korea, Seoul, South Korea; 7 Department of Biomedical Science, College of Medicine, The Catholic University of Korea, Seoul, South Korea

**Keywords:** Tissue engineering, Biomedical engineering

## Abstract

In recent tracheal tissue engineering, limitations in cartilage reconstruction, caused by immature delivery of chondrocyte-laden components, have been reported beyond the complete epithelialization and integration of the tracheal substitutes with the host tissue. In an attempt to overcome such limitations, this article introduces a protective design of tissue-engineered trachea (TraCHIM) composed of a chitosan-based nanofiber membrane (CHIM) and a 3D-printed biotracheal construct. The CHIM was created from chitosan and polycaprolactone (PCL) using an electrospinning process. Upon addition of chitosan to PCL, the diameter of electrospun fibers became thinner, allowing them to be stacked more closely, thereby improving its mechanical properties. Chitosan also enhances the hydrophilicity of the membranes, preventing them from slipping and delaminating over the cell-laden bioink of the biotracheal graft, as well as protecting the construct. Two weeks after implantation in Sprague–Dawley male rats, the group with the TraCHIM exhibited a higher number of chondrocytes, with enhanced chondrogenic performance, than the control group without the membrane. This study successfully demonstrates enhanced chondrogenic performance of TraCHIM in vivo. The protective design of TraCHIM opens a new avenue in engineered tissue research, which requires faster tissue formation from 3D biodegradable materials, to achieve complete replacement of diseased tissue.

## Introduction

Recent studies in tissue engineering field have focused on soft-tissue regeneration, while research in hard-tissue reconstruction has matured; various biofabrication methods and biomaterials suitable for soft-tissue engineering have been introduced^1–5^. In particular, researchers have attempted cell-based approaches using cell-sheets or three-dimensional (3D) cell-encapsulating scaffolds^6–9^, since due to limited regenerative capacity of cell-free scaffolds^10–12^. However, such cell-based approaches are not expected to achieve efficient tissue regeneration, due to the poor transfer capacity of cell-laden structures. The cell-related characteristics of the structures are regulated by the stiffness of the cell surroundings, which should be < 30 kPa^13,14^. Cell-laden delivery scaffolds with such weak mechanical properties can easily be torn and lost upon handling or become delaminated from their polymeric frames, reducing the regenerative capability of the constructs^14^. Thus, protective methods for the lossless delivery of cell-based constructs are required.

The trachea is a soft tissue supported by C-shaped cartilage and various approaches have been proposed for creating tracheal constructs^15–20^. Although many of them have achieved successful epithelialization in the tracheal lumen and good tracheal integration, cartilage reconstruction remains limited. Since cartilage formation time-consuming (requires regenerative time than any other tissues), recent approaches have focused on the stable delivery of chondrocytes, the metabolically active cells that synthesize a large quantity of the cartilage extracellular matrix (ECM) components, such as collagen, proteoglycans, and hyaluronan. Dennis et al.^15–20^ prepared neotracheal silicon tubes covered with tissue-favorable sheets, including skin grafts, platysma flaps, and cartilage sheets. Good tracheal integration without lumen collapse and epithelization was observed three weeks after implantation in rabbits; however, due to delamination of the tissue layers, minimal cartilage reconstruction was observed. To promote adhesion between the cell-sheets and other layers, Gao and colleagues^15–20^ introduced a pre-vascularized tracheal unit by preoperative implantation of 3D-printed poly-L-lactide (PLLA) scaffolds covered with sheets of chondrocytes^15–20^. However, pre-vascularization accelerated epithelialization only in the second week after transplantation, with little retention of chondrocyte sheets around the scaffolds, referred to as chronic limitation in soft-tissue engineering. To overcome these limitations, protective, high-strength tracheal constructs including chondrocyte encapsulating cartilage part have been designed. Park et al.^15–20^ fabricated multi-layered PCL scaffolds using 3D bioprinting technology; layers of alginate encapsulating epithelial cells and chondrocytes were located separately between the PCL layers. Nonetheless, most chondrocytes disappeared, few left, and little chondrogenic activity was observed.

In this article, we present a new design of a tissue-engineered trachea with a protective chitosan-based nanofiber membrane (TraCHIM) for in vivo reconstruction of cartilage. To design the protective membrane, the following criteria were considered: the membrane should be (i) biocompatible and biodegradable, (ii) flexible to bend and surround the biotrachea, and (iii) hydrophilic, to retain the bioinks and integrate with native tissues. In biomedical applications, electrospun nanofiber membranes have been used due to their high surface-area-to-volume ratio, which provides an abundance of protein absorption sites and numerous cell-binding sites^21^. Among the various biomaterials used to create electrospun membranes^22–24^, chitosan has shown superior biological and physiochemical properties in in vivo implantation; these include high hydrophilicity and non-toxic degradation by-products compared with those of other polymeric materials^25^. Therefore, electrospun chitosan-based nanofiber membrane (CHIM) was introduced for tracheal protection. CHIM, which consists of PCL and chitosan, was fabricated via electrospinning. To demonstrate the efficacy of CHIM, we measured the water contact angle, diameter, and mechanical properties of the electrospun fibers. Thereafter, we assessed TraCHIM in vivo by their ectopic subcutaneous implantation into rats. At two weeks after implantation, we observed the differences in pore size by assessing the degradation of the polymeric frame of biotracheal grafts and the presence of surviving chondrocytes, which indicated cartilage regeneration. Our study demonstrates that these protective tissue-engineered tracheal grafts, TraCHIM, display enhanced chondrogenic performance in vivo and have potential for clinical use in future.

## Results and discussion

### Protective design of a bioengineered trachea

**Figure 1:**
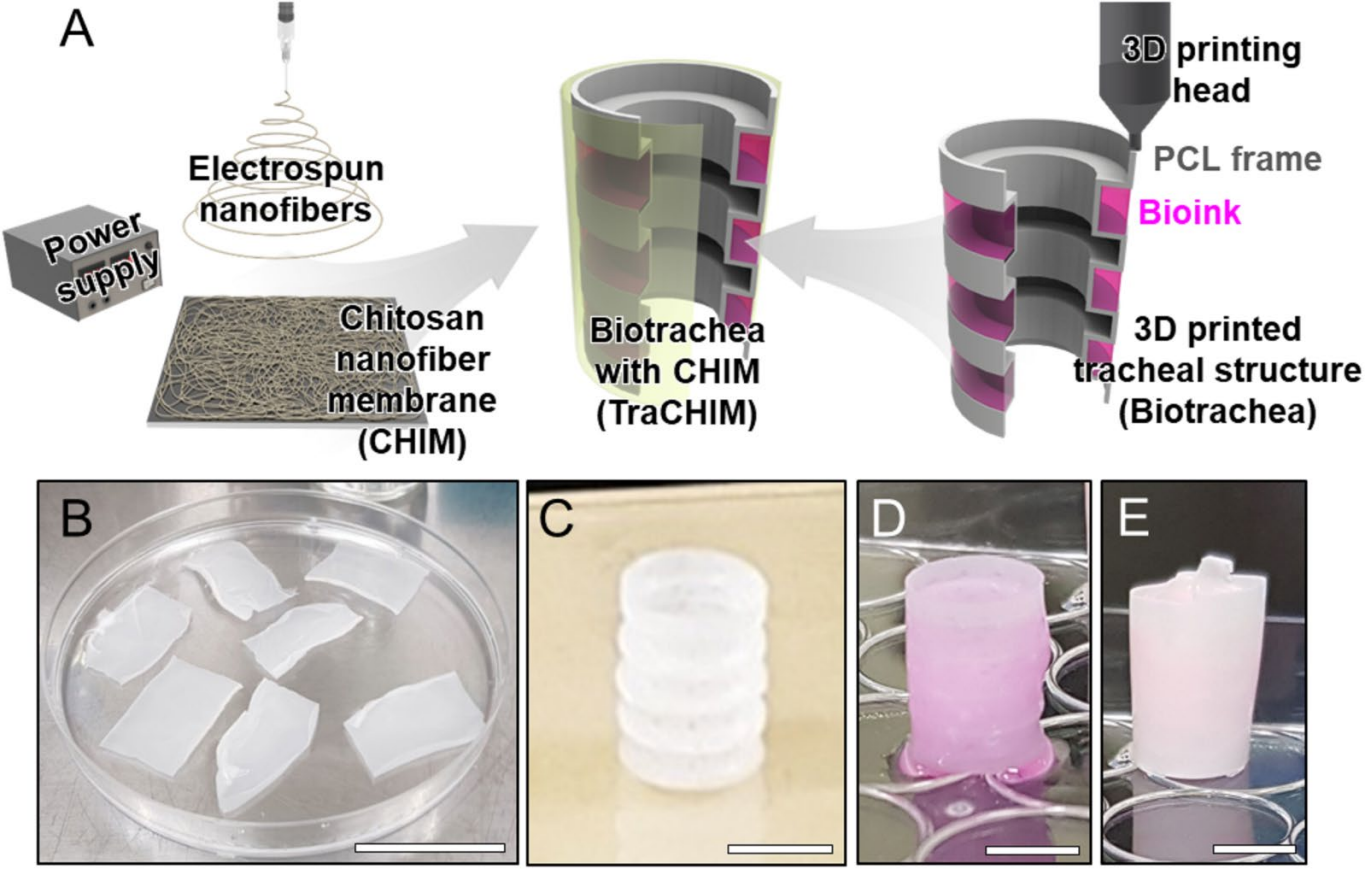
(**A**) Schematic of the fabrication of a 3D-printed tracheal structure (biotrachea) with an electrospun chitosan-based nanofiber membrane (CHIM). Photographs of (**B**) CHIM, (**C**) 3D-printed PCL frame, (**D**) the biotrachea, and (**E**) the biotrachea with CHIM (TraCHIM). Scale bars, 5 cm, 5 mm, 5 mm, and 5 mm, respectively.

Given the limitations of cartilage reconstruction in tracheal tissue engineering, we developed a protective membrane for biotracheal constructs to support bioink retention during the early postoperative days, thereby facilitating cartilage regeneration (Fig. 1A). This membrane, named CHIM, is an electrospun nanofiber sheet created from chitosan and PCL (Fig. 1B). The 3D-printed biotrachea consists of a porous PCL bellows framework and bioink, which is a biocompatible collagen hydrogel encapsulating chondrocytes (Fig. 1C,D). To protect the bioink promoting cartilage reconstruction, CHIM was assembled to surround the biotracheal graft (Fig. 1E).

### Characteristics of the CHIM

**Figure 2:**
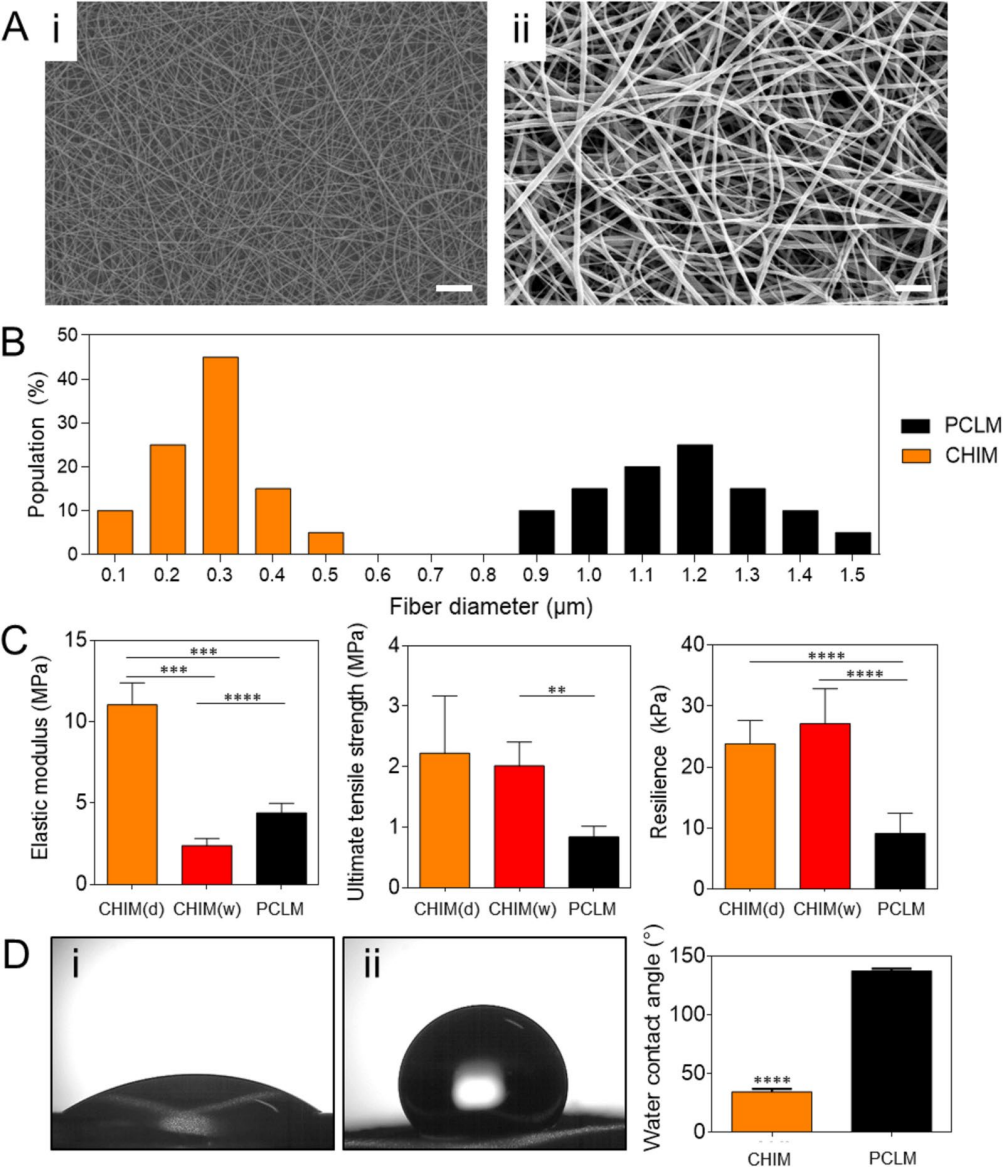
Characteristics of the chitosan-based nanofiber membrane (CHIM): (**A**) Scanning electron microscopy (SEM) images of the membranes at 1000 × magnification: (i) CHIM and (ii) Polycaprolactone (PCL)-based membrane (PCLM). Scale bars, 10 μm. (**B**) Distribution of fiber diameters based on the SEM images. (**C**) Mechanical properties of CHIM and PCLM in the dry and wet states. (**D**) Water contact angle of each membrane: (i) CHIM and (ii) PCLM. Each bar reflects the mean ± standard deviation. **p < 0.01, ***p < 0.005, ****p < 0.001.

The CHIM was fabricated by electrospinning a solution of chitosan and PCL (weight ratio of 1:3) and investigated using Fourier-transform infrared spectrophotometer (FTIR; VERTEX 70, Bruker, Germany), as shown in Fig. S1. As a control, a PCL-based nanofiber membrane (PCLM) was prepared. Scanning electron microscopy (SEM) revealed that the inner structures of both the PCLM and CHIM contained isotropic random nanofibers (Fig. 2A). Although both the PCLM and CHIM were fabricated under the same electrospinning conditions, their respective fiber diameter of 1.17 ± 0.16 μm and 0.28 ± 0.10 μm differed significantly (Fig. 2B). The lower fiber diameter of CHIM may be attributed to the chitosan, which changes both the viscosity and charge density of the electrospinning solution^26^. A higher conductivity of electrospinning solution enhances charge transport by the polymer jet during electrospinning, thereby stretching the polymer jet and resulting in a thinner fiber diameter^27^.

Interestingly, the change in fiber diameter affected the mechanical properties of membranes (Fig. 2C). The measured elastic moduli of the PCLM and CHIM were 4.39 ± 0.48 MPa and 11.05 ± 1.10 MPa, respectively. Furthermore, intermixing with chitosan (in the CHIM) improved the ultimate tensile strength and resilient modulus of the PCLM by 2.64 and 2.60 times, respectively. This inverse relationship between the fiber diameter and elastic modulus could be attributed to the strong interactions of the macromolecular chains in the thin fibers^28,29^. In addition, considering that the elastic modulus of CHIM is similar to that of in vivo native tracheal cartilage (of the order of 10 MPa)^30^, CHIM is a potential tracheal substitute.

Another important characteristic of the nanofiber membrane useful for practical use in vivo is its surface wettability. Although PCL is a biodegradable and biocompatible polymer with good mechanical properties, additional treatments are necessary to reduce its hydrophobicity. Poor surface wettability inhibits the cellular adhesion on the membrane and causes the membrane to delaminate from tissues *in vivo*^15–20^. Blending hydrophilic chitosan with PCL overcomes these problems. Comparing the measured contact angles of CHIM (34.5°) and PCLM (137.3°) revealed the greater surface wettability of CHIM (Fig. 2D). This improved surface wettability enables the CHIM to maintain contact with the bioink, and thereby prevent the membrane from slipping over the bioink. Furthermore, the enhanced hydrophilicity of the CHIM reduces the bioinertness and encourages in vivo integration.

### In vivo implantation

**Figure 3:**
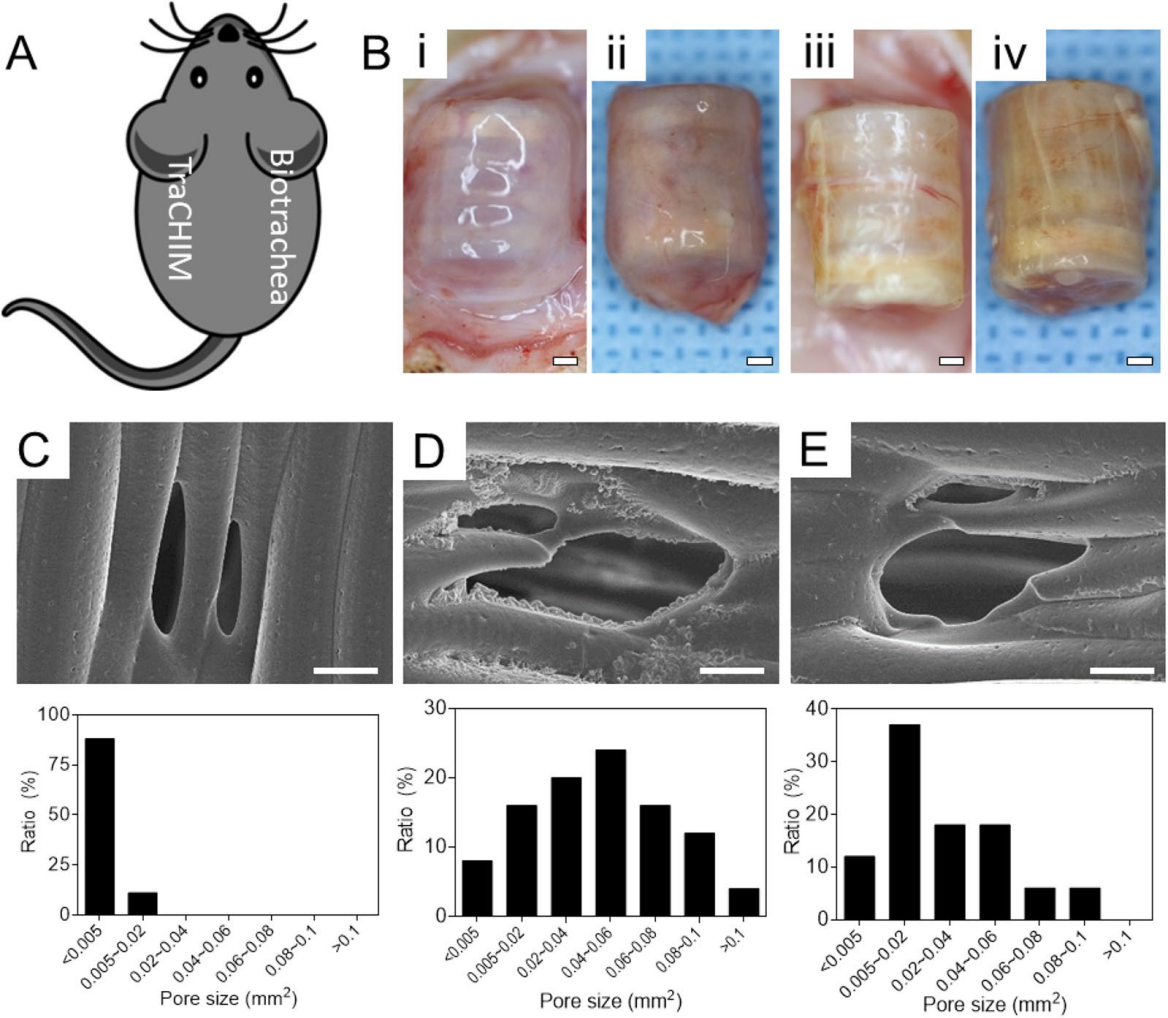
In vivo assessment: (**A**) Dorsal implantation site in rats. (**B**) Images of the harvested samples in post-operative 2 weeks: Biotrachea (i) in vivo and (ii) post-harvest; TraCHIM (iii) in vivo and (iv) post-harvest. Scale bars, 1 mm. SEM images (× 200) and pore distributions of post-harvest biotracheal PCL frames: (**C**) Original PCL frames; (**D**) Biotrachea-only group; and (**E**) TraCHIM group. Scale bars, 100 μm.

**Figure 4:**
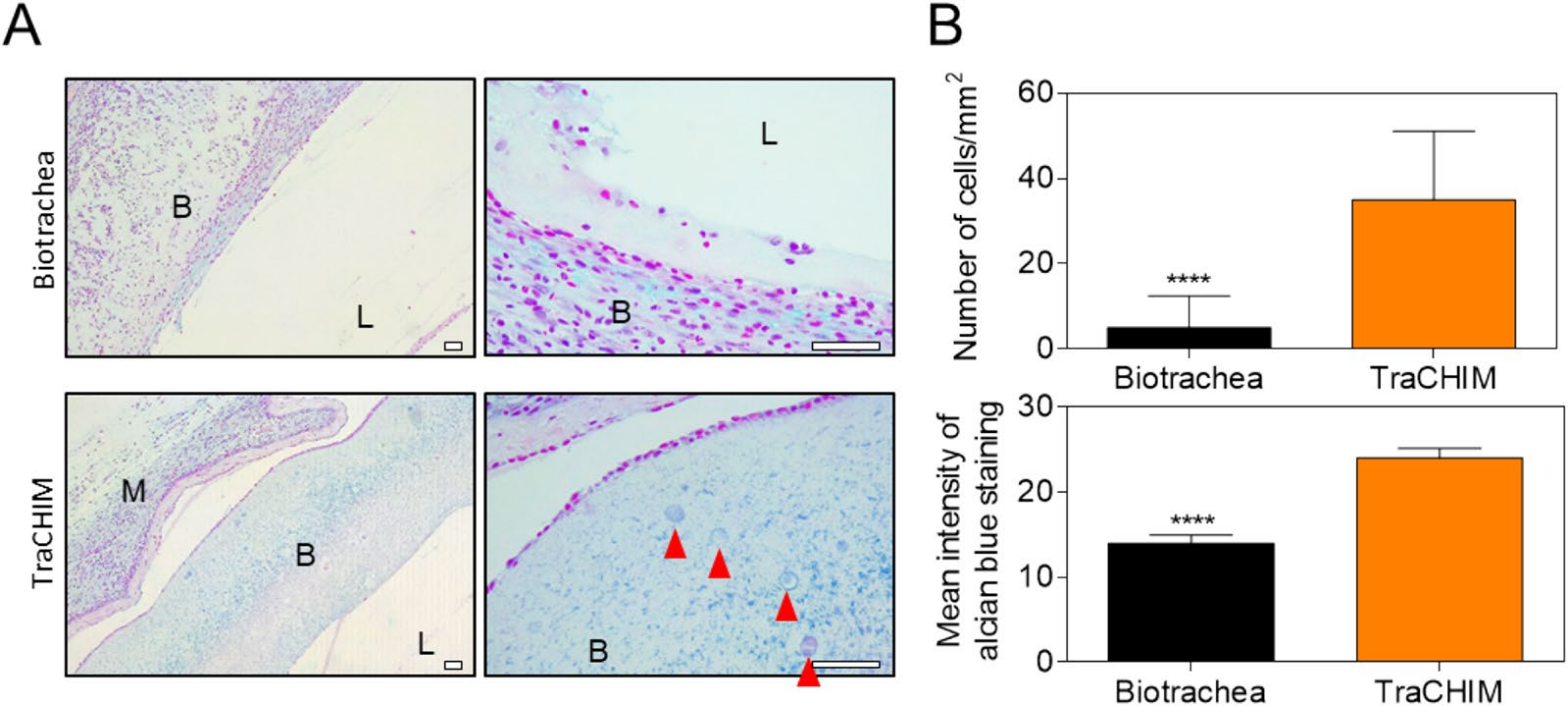
(**A**) Alcian blue-stained histological images of tissues harvested from the biotrachea- and TraCHIM-implanted groups. B, L, and M: bioink, lumen, and membrane, respectively. Red arrows indicate encapsulated chondrocytes. Scale bars, 50 μm. (**B**) Numbers of chondrocytes per unit area, and the mean intensity of alcian blue staining, in the histology images. Bars reflect the mean ± standard deviation. ****p < 0.001.

We assessed the effectiveness of the biotracheal graft assembled with a protective membrane (TraCHIM) via in vivo implantation. Two experimental biotracheal implants were used: (i) TraCHIM, prepared by assembling CHIMs and biotracheal constructs, and (ii) a biotracheal construct (the control) that comprised a PCL frame and bioink. The constructs were fabricated using 3D-printing technology. The constructs were implanted subcutaneously in rats and the outcomes were observed after two weeks. None of the implanted grafts displayed changes in structure, nor an inflammatory response was observed during the postmortem examination. The tensile moduli of the implanted grafts of both the TraCHIM and the control groups were of the same order of magnitude as that of the native trachea (10.6 ± 1.8 MPa) (Fig. S2)^31^. These results indicated that the biotracheal construct and CHIM reduced the risk of tracheal collapse, which is generally occurred due to the differences in mechanical properties between the native trachea and implanted grafts^32^. In addition, the PCL frame degraded in both groups and the TraCHIM resulted in smaller pores than the control (Fig. 3). Previous tissue-engineered tracheas were based on PCL framework due to its biocompatibility and biodegradability (~ 2 years to degrade)^33–35^. However, body fluids in vivo accelerate PCL degradation, which reduces the structural support required to regenerate tracheal tissue. The different pore sizes indicate that the CHIM reduced the in vivo degradation rate of the tracheal frame, thereby safely retaining the delivered bioinks within the TraCHIM. Histological examinations revealed that the chondrocytes were better maintained and secreted more glycosaminoglycans (GAGs) in the TraCHIM group than in the control group (Fig. 4).

Recent research on cartilage reconstruction has highlighted the long time required for cartilage formation and the need for improved integration in vivo. Unfortunately, the chondrocytes and chondrocyte precursors on trachea are easily absorbed in the host tissue, or lost by delamination from the constructs^15–20^. Our design presents the possibility of overcoming these limitations via CHIM use in vivo as CHIM helps to maintain the tracheal cartilage component of the biotracheal constructs, which stabilize chondrocytes by conferring protection against any stimuli upon transplantation and delaying the rapid in vivo degradation and absorption of medical grade PCL.

## Conclusion

We have successfully demonstrated the potential of a protective tissue-engineered tracheal graft, with enhanced in vivo chondrogenic performance, for tracheal tissue engineering. Although these grafts improved short-term cartilage regeneration, long-term tracheal implantation studies are required to evaluate the efficacy of tracheal substitutes for regenerating mature cartilage. This protective design provides a blueprint for cartilage regeneration and will be applicable in tissue engineering research for developing matured tissue analogs.

## Experimental section/methods

### Fabrication of biotracheal construct

The experiments on human nasal septum-derived chondrocytes were performed in compliance with the Institutional Review Board of the Catholic Medical Center Clinical Research Coordinating Center (KC08TISS0341), and the written informed consents from the donors were obtained. The chondrocytes were isolated as previously described^36^. The chondrocytes (5 × 10^6^ cells/mL )were then mixed with collagen gel (2.7% w/v; Ubiosis, Korea).

The PCL frame, as the basic structure of the biotracheal scaffold, was 3D-printed based on the previously designed bellows structure^15–20^. PCL pellets (molecular weight 43–50 kDa; Polyscience Inc., USA) were prepared and extruded via a 90 °C printing head using the in-house 3D-printing system. The bellows-type framework had following dimensions: length 13 mm; internal diameter 7 mm; pore size 200 µm; and wall thickness 400 µm. The chondrocyte-encapsulated collagen was 3D-printed in each groove of the bellows. Completed biotracheal grafts were cultured *in vitro* for 5 d before implantation.

### Fabrication of tracheal construct and chitosan membrane

Electrospinning solutions for chitosan (High molecular weight: 310–375 kDa; Sigma-Aldrich, USA) and PCL (M_n_ 80,000 g/mol; Sigma-Aldrich, USA) were prepared separately. Chitosan (12.5% w/v) was dissolved in a mixed solvent containing trifluoroacetic acid (TFA; 99%; Sigma-Aldrich, USA) and dichloromethane (DCM, > 99.8%; Sigma-Aldrich, USA) at a volume ratio of 7:3, followed by stirring at 50 °C for 6 h. PCL pellets (at 7.5% w/w) were dissolved in 2,2,2-trifluoroethanol (TFE, 99%; Alfa Aesar, USA) and stirred at 25 °C (room temperature) for 10 h.

To fabricate CHIM, the chitosan solution and PCL solution were blended at a weight ratio of 1:3. Due to acidic hydrolysis and degradation of PCL caused by TFA, the blended solution should be prepared just before electrospinning; the blended solution retains its electrospinnability for up to 1 h after preparation^37^. The blended solution was loaded into a gastight syringe (Hamilton, USA) and ejected through a 21-gauge needle at a constant flow rate (1.5 mL/h) using a syringe pump (KDS200, KD Scientific, USA). Next, a high voltage (19 kV) was applied between the metal capillary and a metal collector using a power supply (HV30, NanoNC, Korea) at a vertical distance of 14 cm. During electrospinning for 20–30 min, a relative humidity of 50–60% and a temperature of 20–25 °C were maintained. The thickness of the fabricated membranes was within 40–50 µm, as measured using a micrometer (Mitutoyo, Japan). PCLM was fabricated using the same electrospinning conditions, by loading the PCL solution into the gastight syringe.

### Electrospun nanofiber membrane treatment for in vivo implantation

Before in vivo implantation, the CHIM need to be neutralized and sterilized^38^. The prepared CHIM was desiccated for 24 h to evaporate residual toxic solvent, then neutralized in ammonium hydroxide (14% by weight) (OCI Company, Korea) for 15 min and rinsed with deionized water three times to remove the remaining ammonium hydroxide. Then, the CHIM was then sterilized using 70% ethanol for 15 min and in fresh 70% ethanol for an additional 45 min, sequentially. The CHIM was rinsed with deionized water three times for three min each, followed by rinsing with phosphate-buffered saline (PBS, Hyclone, USA; pH 7.4) three times for three min each. Subsequently, the CHIM was immersed in fresh PBS overnight at 4 °C. Prior to in vivo implantation, the CHIM was transferred to fresh PBS.

### Measurement of contact angle

To investigate the surface wettability of the electrospun nanofiber membranes, 10 μL of deionized water was placed on the membrane. The SmartDrop device (Femtobiomed, Korea) was used to measure the static contact angle of the sessile water droplet on the membrane surface at room temperature.

### Quantification of fiber diameter

To examine the structure of the electrospun membranes, the membranes were dried, and sputter coated with Au–Pd at 10 mA for three min. SEM (FE-SEM SU6600, Hitachi, Japan) images of the membrane were obtained at an accelerating voltage of 15 kV. The membrane fiber diameter was quantified from the SEM images using ImageJ software (NIH, USA).

### Mechanical properties

Mechanical testing was performed using the two methods depending on the products as follows: (i) The electrospun membrane was trimmed into a dog-bone-shaped specimen using a laser cutter. The specimen, which was downsized based on the ASTM D638 standards, had a 6 mm gauge length, 4 mm width, and 50 µm thickness. Each end of the specimen was gripped in a clamp and its tensile properties was tested at a constant tensile speed of 10 mm min^−1^, using a customized testing machine comprising a linear actuator and a load cell with a resolution of 0.01 gf. The resultant force–distance curve was converted to a stress–strain curve for evaluating the elastic modulus, ultimate tensile strength, and resilience. (ii) To investigate the mechanical behavior of the 3D-printed biotracheal construct, a single-column Instron 3340 mechanical testing system (Instron, Norwood, MA, USA) was used. Mechanical testing of radial compression was performed at a cross-head velocity of 0.5 mm min^−1^ with monitoring of the load and displacement. The resultant force-distance curve was converted to a stress–strain curve to analyze the compressive modulus.

### In vivo assessment

Six Sprague–Dawley male rats (approximately 300–450 g, approximately 8–9 months old) were used for the in vivo study. All experiments were approved by the Institutional Animal Care and Use Committee (IACUC) at POSTECH and performed in strict accordance with the recommendations in the Guide for IACUC guidelines (IACUC permit No. POSTECH-2018-0031). In addition, the study was carried out in compliance with the ARRIVE guidelines. Before implantation, the TraCHIMs were manually prepared by rolling the 3D printed biotracheal constructs on the CHIM. The rats were categorized into two groups (n = 5 each): the first group received a biotrachea scaffold only (control), while the second group a biotrachea scaffold surrounded by the CHIM. The rats were anesthetized using isoflurane (induced at 4% and maintained at 2%), shaved, and administered subcutaneous injection of buprenorphine (0.6 mg kg^−1^). The animals were positioned in sternal recumbency for dorsal implantation. After scrubbing with betadine, dorsal side incisions (10 mm) were made over the posterior region. Twelve scaffolds were inserted subcutaneously on both sides.

### Histological examination

Two weeks after implantation, the rats were sacrificed according to the euthanasia guidelines adapted from the Veterinary Medical Association Guidelines for the Euthanasia of Animals. Harvested samples were embedded in paraffin and slides were stained with hematoxylin and eosin and Alcian blue. The stained specimens were visualized using a microscope. Tracheal structure, chondrocyte density, and inflammatory cell infiltration were evaluated by a pathologist blinded to the treatment modalities. Chondrocyte density was quantified as the number of chondrocytes in three randomly selected fields per slide viewed at 10 × magnification. The intensity of Alcian blue staining was measured using ImageJ software.

### Distribution of pores on tracheal construct

To observe morphological changes in the 3D-printed scaffolds during degradation, the surface structures and pore sizes were observed using SEM (FE-SEM SU6600, Hitachi, Japan). Pore size was measured using ImageJ software.

### Statistical analysis

All statistical data are expressed as mean ± standard deviation. Data were analyzed using two-way analysis of variance followed by post-hoc Tukey test. Results with *p* < 0.05 were considered statistically significant.

### Study approval

All procedures involving human subjects were approved by the Institutional Review Board of the Catholic Medical Center Clinical Research Coordinating Center (KC08TISS0341) and conducted in accordance with relevant guidelines and regulations^15–20^. Investigations were conducted according to the principles expressed in the Declaration of Helsinki, and the written informed consents from the donors were obtained. All animal experiments were approved by the Institutional Animal Care and Use Committee (IACUC) at POSTECH and performed in strict accordance with the recommendations in the Guide for IACUC guidelines and regulations (IACUC permit No. POSTECH-2018-0031).

## Supplementary Information

Supplementary Information
