# Supplementary material for: Improved chondrogenic performance with protective tracheal design of Chitosan membrane surrounding 3D-printed trachea
Source: Sci Rep. 2021 Apr 29;11:9258. doi: 10.1038/s41598-021-88830-3 (PMC8085235; doi:10.1038/s41598-021-88830-3)
Supplement: Supplementary file 1 — Supplementary Information [file 41598_2021_88830_MOESM1_ESM.docx]

**Supplementary information**

**Full title:** Improved Chondrogenic Performance with Protective Tracheal Design of Chitosan Membrane Surrounding 3D-printed Trachea

**Authors:** Hyeonji Kim^†,a^, Jae Yeon Lee^†,b,^, Hyeonseok Han^†,a^, Won-Woo Cho^a^, Hohyeon Han^c^, Andrew Choi ^a^, Hyeonjun Hong ^a^, Jae Yun Kim^c^, Jeong Hun Park^e^, Sun Hwa Park^f,g^, Sung Won Kim^f,g^, Dong Sung Kim^a^*, Dong-Woo Cho^a,c,d^*

^†^H. Kim, J. Y. Lee, and H. Han contributed equally to this work.

^a^Department of Mechanical Engineering, Pohang University of Science and Technology (POSTECH), 37673, Pohang, Gyeongbuk, South Korea

^b^Department of Companion Animal Health, Daegu Haany University, 38610, Gyeongsan, Gyeongbuk, South Korea

^c^School of Interdisciplinary Bioscience and Bioengineering, POSTECH, 37673, Pohang, Gyeongbuk, South Korea

^d^Institute of Convergence Science, Yonsei University, 03722, Seoul, South Korea

^e^The Wallace H. Coulter Department of Biomedical Engineering, Georgia Institute of Technology and Emory University, Atlanta, GA, USA

^f^Department of Otolaryngology and HNS, College of Medicine, The Catholic University of Korea, Seoul, South Korea

^g^Department of Biomedical Science, College of Medicine, The Catholic University of Korea, Seoul, South Korea

Corresponding authors:

Prof. Dong-Woo Cho

E-mail: [dwcho@postech.ac.kr](mailto:dwcho@postech.ac.kr)

Prof. Dong Sung Kim

[smkds@postech.ac.kr](mailto:smkds@postech.ac.kr)

**

**

**Figure S1.** Chemical structure of CHIM based on FTIR analysis.

Chitosan-based nanofiber membrane was examined using Fourier-transform infrared spectrophotometer (FTIR; VERTEX 70, Bruker, Germany) with a resolution of 4 cm^−1^ within the range of 500–4000 cm^−1^. The chitosan and PCL polymers were bonded chemically and analyzed by FTIR. The two peaks related to amide and amine groups of chitosan-based nanofiber membrane were detected at 1648 cm^-1^ and 1560 cm^-1^.


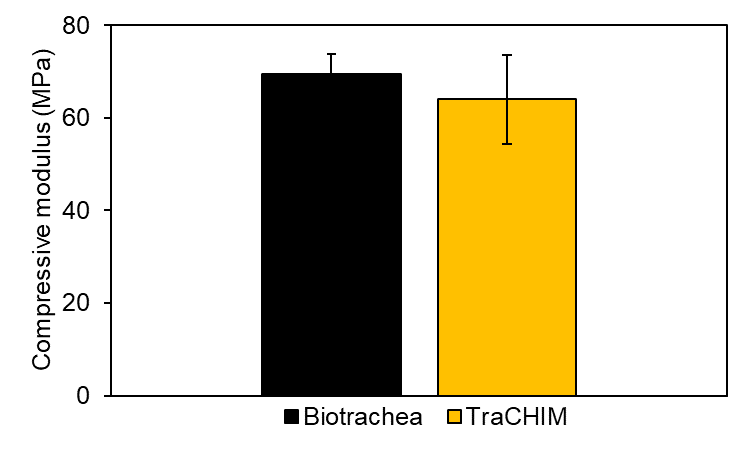


**Figure S2.** The compressive modulus of the implanted grafts
